# Prospective changes of cardiac iron and function in low and intermediate-1 risk mds patients

**DOI:** 10.1186/1532-429X-17-S1-P376

**Published:** 2015-02-03

**Authors:** Alessia Pepe, Antonella Meloni, Giovanni Carulli, Esther N Oliva, Francesco Arcioni, Sergio Storti, Mari Giovanna Neri, Emanuele Grassedonio, Stefania Renne, Gennaro Restaino, Vincenzo Positano, Michele Rizzo

**Affiliations:** 1 CMR Unit, Fondazione G Monasterio CNR-Regione Toscana, Pisa, Italy; 2 Dipartimento di Oncologia, dei Trapianti e delle Nuove Tecnologie in Medicina - Divisione di Ematologia - Facoltà di Medicina e chirurgia, Università degli Studi di Pisa, Pisa, Italy; 3 U.O. di Ematologia, Azienda Ospedaliera Bianchi Melacrino Morelli, Reggio Calabria, Italy; 4 Dipartimento di Medicina Clinica e Sperimentale, Universitaà degli Studi di Perugia, Sez. Ematologia ed Immunologia Clinica, Ospedale "Santa Maria della Misericordia", Perugia, Italy; 5 UOC di Onco-Ematologia,, Università Cattolica del Sacro Cuore - Centro di Ricerca e Formazione ad Alta Tecnologia nelle Scienze Biomediche - "Giovanni Paolo II", Campobasso, Italy; 6 Istituto di Radiologia, Policlinico "Paolo Giaccone", Palermo, Italy; 7 Struttura Complessa di Cardioradiologia-UTIC, P.O. "Giovanni Paolo II", Lamezia Terme, Italy; 8 Dipartimento di Radiologia, Università Cattolica del Sacro Cuore - Centro di Ricerca e Formazione ad Alta Tecnologia nelle Scienze Biomediche - "Giovanni Paolo II", Campobasso, Italy; 9 Reparto di Ematologia, Azienda Sanitaria Provinciale Caltanissetta - Ospedale "Sant'Elia", Caltanissetta, Italy

## Background

In patients with myelodysplastic syndrome (MDS) no longitudinal studies on cardiac iron, function and fibrosis are available in literature.

So, the aim of our study was to assess the changes in cardiac iron overload and in the morpho-functional cardiac parameters by Magnetic Resonance Imaging (MRI) in MDS patients enrolled in the MIOMED (Myocardial Iron Overload in MyElodysplastic Diseases) study who performed the follow-up (FU) MRI at 12 months.

## Methods

MIOMED is an observational, MRI multicentre study in low and intermediate-1 risk MDS patients who have not received regular iron chelation therapy. Out of the 51 MDS patients enrolled, 48 underwent the baseline MRI exam and 28 performed the MRI FU. This analysis was limited to patients who performed both the MRIs. Mean age was 72.8±7.6 years and 8 patients were females. MIO was assessed using a multislice multiecho T2* approach. Biventricular function parameters were quantified by cine sequences. Myocardial fibrosis was evaluated by late gadolinium enhancement (LGE) acquisitions.

## Results

The FU MRI was not performed for the following reasons: 4 deaths and 16 patient refusal.

At baseline only one patient showed cardiac iron overload (global heart T2*=14.8 ms) but he recovered at the FU (global heart T2*=28.8 ms). He was not transfused. Out of the 27 patients without significant cardiac iron at the baseline, 26 maintained the same status at the FU while one showed cardiac iron (global heart T2*=12.3 ms).

Due mainly to technical reasons, biventricular function was assesses at both baseline and FU MRIs in 22 patients. At baseline 6 patients showed a reduced left ventricular ejection fraction (LVEF) and 4 of them recovered at the FU. All patients had a baseline global heart T2*>20 ms (one with 2 segmental T2* values<20 ms). At baseline 5 patients showed a reduced right ventricular EF (RV EF) and all recovered at the FU. One patient with normal LV EF at baseline showed pathological LV EF at the and 2 patients with normal RV EF at baseline showed reduced RV EF at the FU (one patient suffered from pulmonary hypertension). At the FU we detected a significant increase in the LV end-diastolic volume index (EDVI) (mean difference: 6.5±11.3 ml/m2; P=0.015) as well as in the RV EDVI (mean difference: 7.8±9.3 ml/m2; P=0.002). The change in the LV mass index between the 2 MRIs was not significant.

For 18 patients the presence of myocardial fibrosis was investigated at both baseline and FU MRIs, and this subgroup was considered. Eight patients had myocardial fibrosis at the baseline. One patient showed a subendocardial ischemic pattern and seven patients showed a non-ischemic pattern and myocardial fibrosis was detected for all of them also at the FU. At the FU one new occurrence of non-ischemic myocardial fibrosis was detected.

## Conclusions

The new occurrences of cardiac iron, reduced cardiac function, increased LV and RV EDVI and myocardial fibrosis suggest the need of performing periodic MRI scans, in order to better manage these patients.

## Funding

The MIOMED project receives "no-profit support" from industrial sponsorships (Novartis).

**Figure 1:**
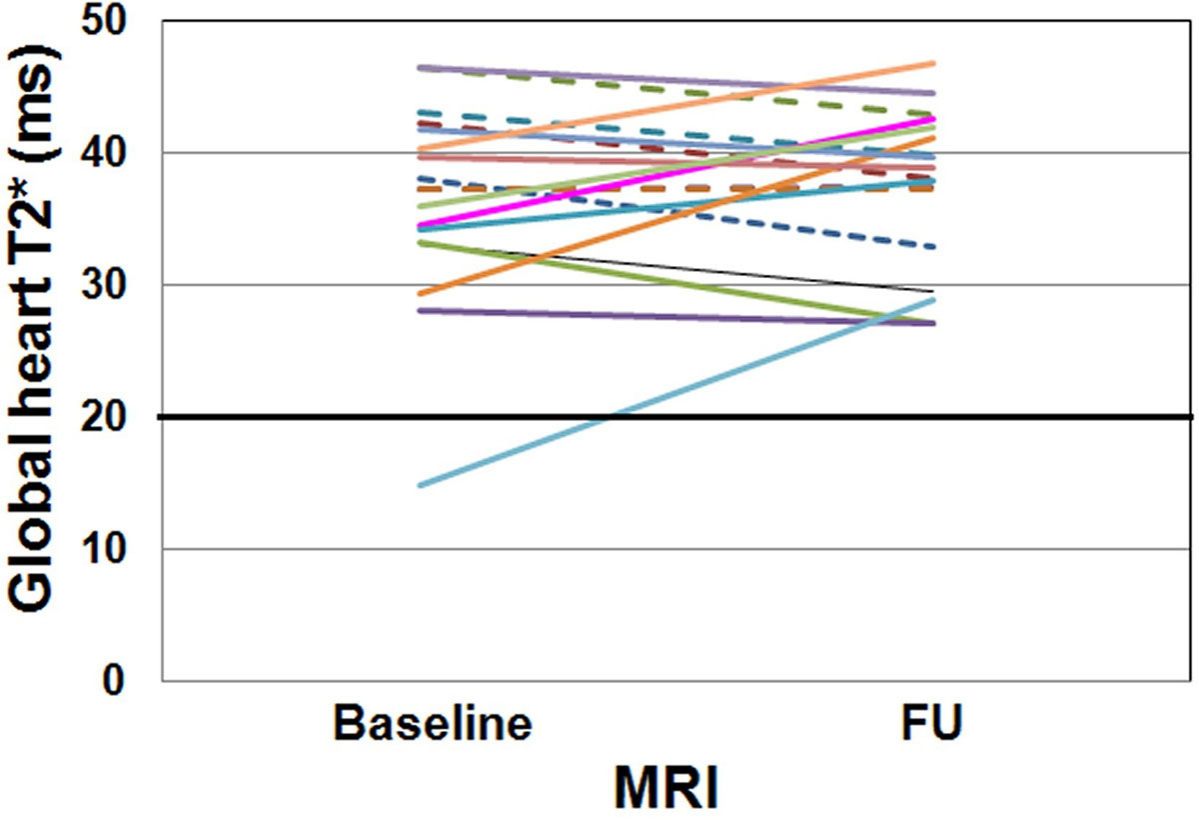
Intraindividual course of the global heart T2* value at baseline and at follow-up.

**Figure 2:**
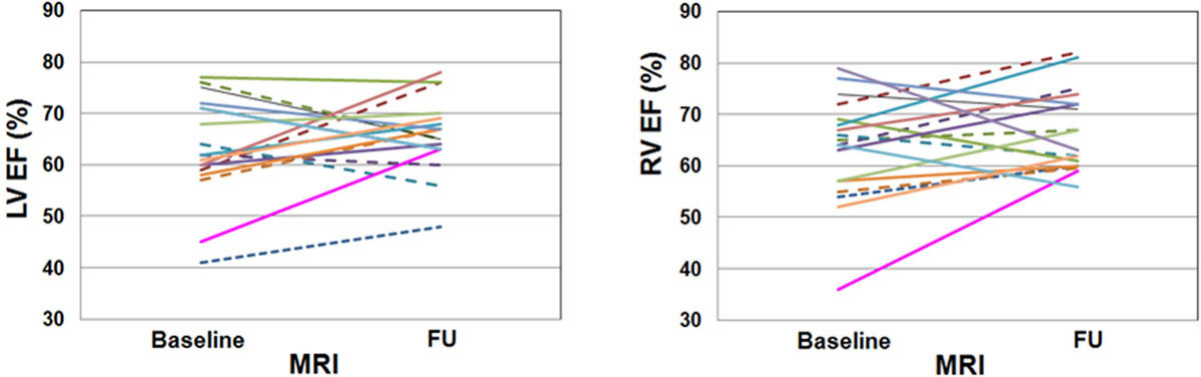
Intraindividual course of LV and RV ejection fractions at baseline and at follow-up.

